# Research trends in the field of sport impact on the economy: a bibliometric analysis

**DOI:** 10.3389/fspor.2025.1545264

**Published:** 2025-05-07

**Authors:** Marina Kudinska, Irina Solovjova, Žanete Korde

**Affiliations:** ^1^ Department of Finance and Accounting, University of Latvia, Riga, Latvia; ^2^ Department of Health Psychology and Pedagogy, Riga Stradinš University, Riga, Latvia

**Keywords:** bibliometric, analysis, sports, impact, publications, scopus

## Abstract

In this paper, the authors summarize the results of the bibliometric analysis. The object of the analysis is scientific publications published in the Scopus database in the scientific field of the impact of sports on the economy. The study aims to fill the research gap in the bibliometric analysis of the impact of sports on the economy by providing an empirical contribution that reveals trends in the scientific literature on the impact of sports on the economy, the most productive researchers, institutions, countries, journals in this field of research, and identifying a bibliometric framework that includes networks between researchers. Scientific articles indexed in Scopus were analyzed with no specific time limits using bibliometric analysis methods—performance analysis, citation analysis, and science mapping. We employed performance analysis, citation analysis, and science mapping via the Bibliometrix package R Studio® and the VOSviewer. The results of the systematic review show that, according to the Scopus database, 801 authors have studied the impact of sports on the global economy, and 299 scientific articles have been published in various journals around the world during the study period. This relatively low number suggests insufficient attention on the part of researchers to the importance of the sports sector. The most active researchers are from the USA, the UK, and China. The most influential journals and research institutions have been identified. The study results showed disagreement between the authors in some areas of the study (economic impact of major sporting events, impact of new sports infrastructure on regional economic growth, illustrating the ongoing debates in the field.

## 1 Introduction

Sport is a growing economic sector that unites businesses, communities, and organizations aiming to foster physical development. The various aspects of the development of the sports industry are the focus of a considerable amount of economic research. For example, the Scopus database used “sport” as a keyword in more than 355,000 studies and the Web of Science database in more than 221,000 studies. Given the rapid growth in the number of publications, the accumulation and organization of information is becoming increasingly complex. Bibliometric analysis, which has become a standard tool for developing a quantitative assessment of academic output in a research field, helps navigate the research and identify research gaps. In bibliometric analysis, publication streams are grouped by criteria such as authors, journals, or countries, offering a quantitative overview of academic output. Bibliometric analysis is a quantitative approach to assessing a body of literature that provides insights into the research environment, influential authors, foundational works, and key research trends. It offers valuable insights into the impact and dissemination of research output, facilitating the identification of influential papers and collaborative networks (1).

The Scopus and Web of Science databases contain several publications on the results of bibliometric analysis of sport-related processes. For example, there are 31 documents in the Scopus database and 26 in the Web of Science database. Bibliometric analysis has been performed on publications in Sports Management (1–6), teaching models of sports (7–9), sports policy (10), several publications have analyzed research activity on issues related to health recovery after injury (11, 12). Bibliometric methods have also been applied to other educational spheres, such as outdoor education (13). However, no publications have been found that have carried out a bibliometric analysis of the impact of sports on the economy. The present study's authors cover this research gap by conducting a bibliometric analysis of the impact of sports on the economy. The authors believe it is essential to study the impact of sports on the economy for several reasons. Firstly, the sports sector is a sector of the economy that itself generates added value; secondly, the sports sector employs companies and organizations that employ personnel, thus increasing the overall employment level of the country; thirdly, the development of the sports sector has an impact on other sectors of the economy, therefore having an indirect effect on the economy (e.g., growth in related sectors such as tourism, retail, and services). In this study, the authors identify how many studies have been carried out worldwide to show the impact of sports on the economy, which aspects of the impact of sports on the economy have received more attention in scientific literature, assess the publication performance of authors and research institutions, and the level of cooperation. The analysis was carried out using bibliometric analysis tools: the Bibliometrix package of R Studio® and the VOSviewer software.

This study aims to fill the gap in bibliometric analyses of sports’ economic impact by providing an empirical review that identifies research trends, the most productive researchers, institutions, countries, and journals. Additionally, we develop a bibliometric framework highlighting authors’ collaborative networks. To achieve the goal, we set out the following tasks: examine bibliometric analysis tools and their functionality, select relevant publications on the impact of sports on the economy using Scopus, perform bibliometric performance and science mapping analyses, and provide conclusions and recommendations based on the results. This study represents the initial stage of research into the influence of sports on the Latvian economy, which is a part of the Latvian state's large-scale research program “Sports.” The next stage will involve assessing the impact of sports on the Latvian economy based on actual data. The experience of different countries, summarized in this study, will contribute to a more objective evaluation of the impact of sports on the Latvian economy.

## 2 Methodology and techniques

VOSviewer and R-studio software were used to gain insight into the research landscape, identify the most influential authors, publication channels, collaboration patterns, conduct citation analysis, and draw conclusions on research trends in the field of the economic impact of sport.

VOSviewer was developed in 2009 to improve the construction and graphical display of bibliometric maps. Its functionality is especially useful for displaying large bibliometric maps in an easy-to-interpret way (14). The Bibliometrix® package developed by Aria and Cuccurullo in 2017 has more functionality for bibliometric analysis than VOSviewer. The proposed tool, which is programmed in R, is flexible and can be quickly updated and integrated with other statistical R packages (15).

**Figure 1:**
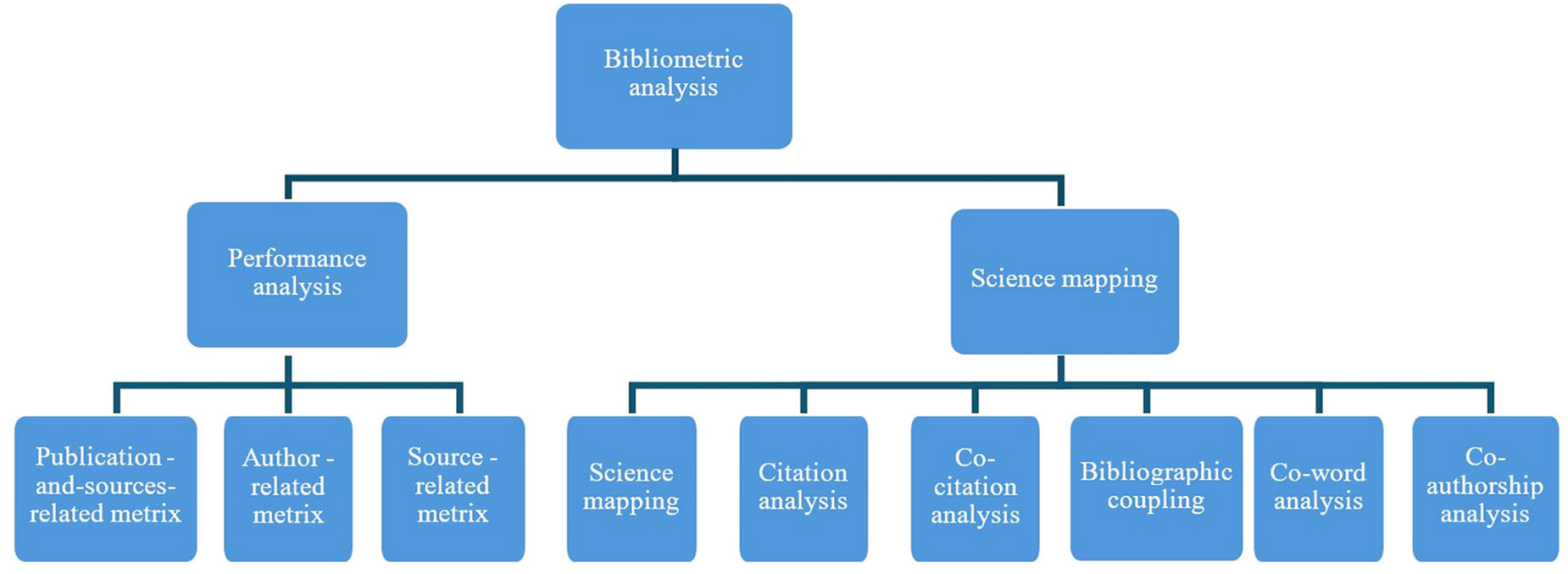
Types of bibliometric analysis.

The bibliometric analysis is carried out according to Donthu et al. (16). In the first stage of the analysis, methods and techniques were selected to perform the analysis. Bibliometric analysis mainly uses two primary methods: performance analysis, which measures productivity and impact, and science mapping, which describes the relationship between publications or their components. In essence, performance analysis accounts for the contributions of research constituents, whereas science mapping focuses on the relationships between research constituents (16) (see Figure 1).

Performance analysis assesses the contribution of authors and co-authors to knowledge through the number of scientific publications. The citation rate describes the impact of previous research on the development of science, including in related fields, which, with a specific citation rate, allows to talk about the emergence of new research directions. The study of collaborations with co-authors enables the assessment of scientific links between scientists, organizations, sectors, including between science and industry, knowledge sectors, and countries. Science mapping explores the relationships between components of a research field (16), and this research uses science mapping techniques such as citation analysis, co-citation analysis, co-author analysis, and keyword analysis. Citation analysis assumes that citations reveal links between publications and identify influential publications in a research field. Co-citation analysis assumes that publications frequently cited together are thematically similar and demonstrate the intellectual structure of the research field and its underlying themes. Co-authorship analysis explores the interactions between authors and their affiliations and describes the social structure of the research field that influences its development. Keyword analysis allows the discovery of topical issues and the prediction of future research topics.

## 3 Data characteristics

Nowadays, numerous services and platforms function as scientific publication databases, providing access to bibliographic information and allowing users to track citation metrics. The largest and most influential of these are Web of Science and Scopus. Each has its specific features, functionality, capabilities, and limitations. For this study, the authors selected publications using identical keywords across both databases—Scopus and Web of Science and found that Scopus covers a broader range of publications. A significant number of publications were indexed in both databases. Therefore, the authors considered the results of the bibliometric analysis based on the Scopus database sufficient for interpreting the scientific scope of the field. Scopus (https://www.scopus.com) is a bibliographic database that offers users a variety of tools for analyzing scholarly articles, including publication counts, citation analysis, co-author metrics, the h-index, impact factor, and more. It indexes more than 22,800 scientific journals, including over 4,200 open-access journals. The authors acknowledge that the total number of publications in all world languages is higher; however, for the purposes of this analysis, only publications in English were included to ensure that the selected articles were relevant to the research topic.

This study uses sources from the Scopus database, which were manually selected by identifying keywords directly related to the topic under study. The retrieved data included scientific articles, proceedings, review articles, book chapters, and early access publications from 1990 to 2024. The search retrieved 299 publications in English. Full articles were used for the analysis to ensure a thorough assessment of the literature on the economic impact of sport. A bibliometric analysis was then carried out using the Bibliometrix® package of R Studio® and VOSviewer®.

The publication data for this study were searched and downloaded from the Scopus database using the search words sport* impact AND economic*. The time range is from 1989 to 2024. 299 documents were selected for analysis: 248 articles, 12 book chapters, 25 conference papers, and 14 reviews. 801 authors’ papers, collaboration and cooperation between authors (Co-Authors per document—2.86%; international co-authorships—19.06%), and collaboration and cooperation between authors (co-authors per document—2.86%; international co-authorships—19.06%) have been analyzed.

The frequency of keywords determines the focus of the research. As Table 1 shows, 1,453 Keywords Plus (Keywords Plus are words or phrases that appear frequently in the titles of references to an article but do not appear in the title of the article itself) and 998 Author's Keywords that authors themselves have indicated in their research were used for the study. Analyzing the Scopus publications with VOSviewer and Bibliometrix, the authors conclude that the most frequently used keywords are “economic impact”, “sustainable development”, “sports tourism”, and “sports events”.

**Table 1:** Key performance indicators for performance analysis.

Description	Results
Timespan	1989:2024
Documents	299
Keywords plus (ID)	1,453
Author's keywords (DE)	998
Authors	
Authors	801
Authors of single-authored docs	73
Authors Collaboration	
Single-authored docs	74
Co-Authors per Doc	2.86
International co-authorships %	19.06
Document Types	
Article	248
Book chapter	12
Conference paper	25
Review	14

**Figure 2:**
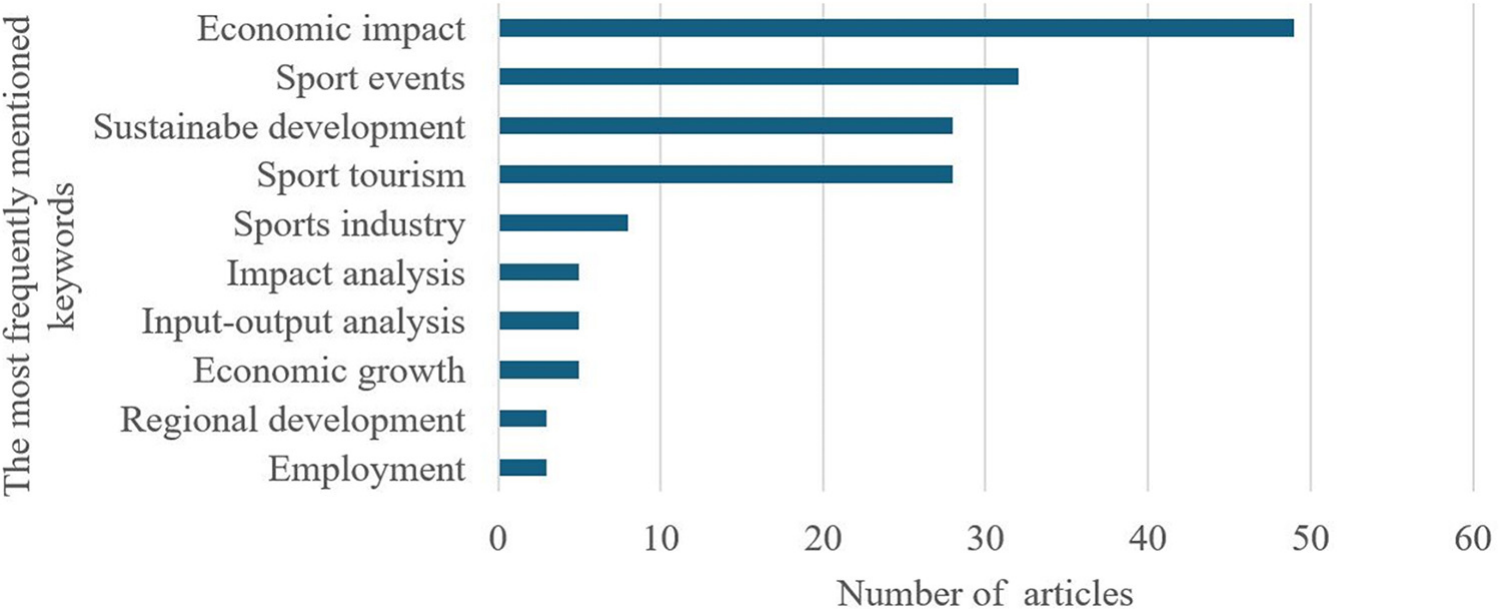
Common keywords in studies on the impact of sports on the economy, according to VOSViewer.

The figure below (see Figure 2) summarizes the number of publications with the ten most frequent keywords in studies on the economic impact of sports and information obtained from VOSViewer.

## 4 Performance analysis

Performance analysis examines the contributions of research constituents to a given field (17). This part of the study analyses the effectiveness of scientific activity in the field of study, the position of countries in the world of science, and the impact of research results on scientific progress.

Performance analysis can be found in most reviews, even in those that do not engage in science mapping, because it is a standard practice in reviews to present the performance of different research constituents (e.g., authors, institutions, countries, and journals) in the field, which is akin to the background or profile of participants that is typically presented in empirical research albeit more analytically (16). As indicated in Figure 1, the performance analysis includes the Publication-and-sources-related metrics, Author-related metrics, and Citation-related metrics.

### 4.1 Publication-related metrics

**Figure 3:**
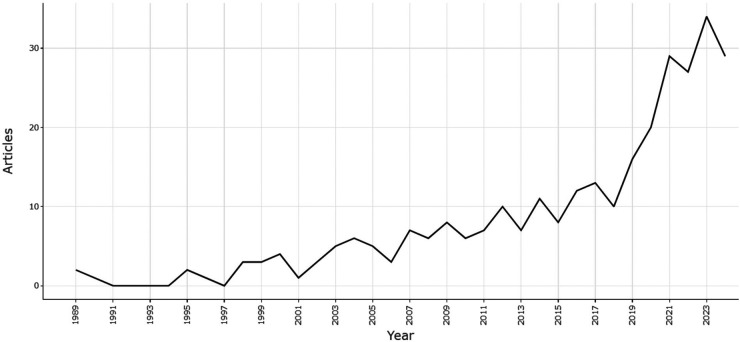
Annual scientific production 1999–2023 according to scopus database data.

The study analyses scientific articles covering a period of 35 years, starting in 1990 when the first study on the economic impact of sports was published in Scopus (18). Figure 3 shows a graph that visually illustrates the dynamics of the number of publications over these years.

Figure 3 shows that until 2017, there was a low number of publications on the researched topic, at around 10 per year. However, since the beginning of 2018, the number of publications has gradually increased, and after 2019, the growth has become significant, which may indicate a rising scientific interest. This increase in publications can be linked to a growing interest in mega-events, sustainable development agendas, additional funding opportunities for scientific projects, and successful international collaboration and cooperation among researchers, universities, scientific centers, etc.

### 4.2 Author-related metrics

There are several authors and researchers who have made significant contributions to the science of the relationship between sports and the economy. According to Scopus, 801 authors have been involved in research on the economic impact of sport. Table 2 below summarizes the most influential authors in this field of study. Information on affiliation is based on the most recent publication. The table uses the following abbreviations: FP—first publication, NP—number of publications, NPF—number of publications fractionalized, h—Hirsh index, g—g-index, m—m-index, TC- total citations. The H-index (Hirsch index) is based on the sum of the most cited papers by researchers and the number of citations they have received in other publications. G-index is introduced as an improvement of Hirsch's h-index to measure the global citation performance of a set of articles. Suppose this set is ranked in decreasing order based on the number of citations they received. In that case, the g-index is the (unique) most significant number such that the top g articles received (together) at least g citations (19). In turn, the m-index is the h-index divided by the number of years of publications under study.

**Table 2:** The most influential authors in the scientific field of the impact of sports on the economy.

No	Author	First publ.	Affiliation	NP	h	g	m	NPF	TC
1	R.A.Baade	1990	Department of Economics and Business, Lake Forest College	5	4	5	0.114	2,17	332
2	V.A.Matheson	2004	Lecturer, Department of Economics and Business, Lake Forest College	5	4	5	0.190	2	218
3	R.W.Baumann	2008	College of the Holy Cross	3	3	3	0.176	1	107
4	B.R.Humphreys	2003	Department of Economics, University of Maryland Baltimore County	3	3	3	0.136	1,33	203
5	J.Zhang	2022	Department of Economics, Clark University	2	2	2	0.667	1	13
7	N.Agha	2012	University of San Francisco	2	2	2	0.167	1,5	70
8	X.Wei	2023	School of Physical Education and Sport Science, Fujian Normal University	1	1	1	0.5	0,25	16
9	R.S. Wu	2009	School of Physical Education, Anhui Normal University	2	1	1	0.063	0,33	15
10	L.E.Pedauga	2024	University of León	1	1	1	0.25	0,33	22
11	C.Navarro	2018	Universidad de Córdoba	2	1	1	0.143	1	12
12	L. Han	2016	Department of Physical Education, China University of Petroleum	2	1	2	0.111	0,67	13
13	S.Zhao	2022	School of Physical Education, West Anhui University	1	1	1	0.333	0,5	3
14	S.P.Crespo	2021	Tecno Campus, Universitat Pompeu Fabra	1	1	1	0.25	0,25	2
15	M.Firgo	2021	Austrian Institute of Economic Research (WIFO)	1	1	1	0.25	1	7

Baade was one of the first researchers to study the impact of sports on the economy, publishing a paper in 1990 (18). The author's subsequent works have also been devoted to the study of the development impact of sport. His research often focuses on whether sports events and infrastructure provide tangible economic benefits to the local economy (20).

The authors of this article would like to highlight one more researcher—Matheson. Matheson is a professor and researcher in sports economics who, in collaboration with Baade, has been actively studying the impact of sports on tourism flows (21) and the economic impact of major sporting events such as the Olympic Games or the World Cup (22–25). He has been critical of public spending on sports infrastructure and analyzed sporting events’ short- and long-term economic benefits. The data in Table 2 indicate that Baade and Matheson have the most publications in the scientific field under study; they have been the most cited authors (highest h-index and highest citation counts) and have the highest citation impact (highest g-index).

Research horizons deepened by Humphreys. Humphreys is a prominent researcher in the field of sports economics. He is well-known for his work on the economic impact of sports, especially concerning the effects of professional sports teams and stadiums on local economies, public funding for sports infrastructure (26), and the broader relationship between sports and economic development. Humphreys has published several scholarly articles exploring the economics of sports leagues, the financial impact of mega sports events (27), and the impact of sports on income and employment (28).

The digital economy is a key driver for the development of the sports sector in line with the Sustainable Development Goals. Zhang et al. (29) have studied the role of the digital economy in the sustainable development of the sports sector and the relationship between the stock market and sports events in the Chinese economy (30).

Agha is a well-known researcher in sports economics, particularly for her work on the impact of small sports leagues, events, teams, and investments on local economies, including job creation, the growth of local businesses, and the overall financial impact on cities hosting sports competitions (31).

The sports sector has become a major contributor to global economic growth, creating employment opportunities and generating revenue through various channels. Wu (32) analyses the multifaceted performance of the sports sector, covering sports-related goods and services, media, tourism, and advertising. The author concludes that there is a positive and significant relationship between sports output and economic growth.

It is worth noting that studies on the economic impact of sports have mainly analyzed a single country. For example, Navarro, Pérez, and Pirela (33) show the impact of sports on economic growth in Colombia. Using econometric modelling, the author has shown that sporting events have a positive impact on employment levels, provide an inflow of investment, and positively impact the income of the entertainment industry and media in Colombia. A similar study by Han (34) on China should be mentioned here.

The hosting of large-scale sporting events not only has a positive impact on the economy and society and has negative consequences (35), such as resource waste and environmental pollution. Zhao and Sun (35) propose to use innovative approaches to impact assessment using neural ticks by building a dedicated model. Experimental results show that the model has high estimation accuracy and is essential for monitoring and managing sporting events.

Sports stimulate the economy and improve social benefits. Crespo (36) uses input/output tables and a cost/benefit analysis methodology to assess a sporting event's economic and social impact. Using data collected from the MedSailing event in 2019, a positive return on every euro invested in the sports event is demonstrated, both economically and socially. Additionally, results related to the media impact of the sports event are presented. A single measure of net return to society allows policymakers to assess the social value of the investment itself and to make decisions that improve the economic development of the area.

The authors would like to draw attention to the study by Firgo (37). This is the first paper to study the impact of hosting the Olympic Games on regional economies. The results show how hosting the Summer Olympic Games increases regional GDP per capita by around 3–4 percentage points in the short term, and the positive long-term effects are also demonstrated. In contrast, the Winter Olympic Games have no positive impact on the host regions and even lead to a decline in GDP per capita in the following years.

Based on the results of the R-studio program, the authors have selected the most relevant studies analyzing different aspects of the economic impact of sport. The authors conclude that there is a trend towards the development of a model that would be able to quantify not only qualitative, but also quantitative impacts on countries, host regions, and individual sectors of the economy.

**Figure 4:**
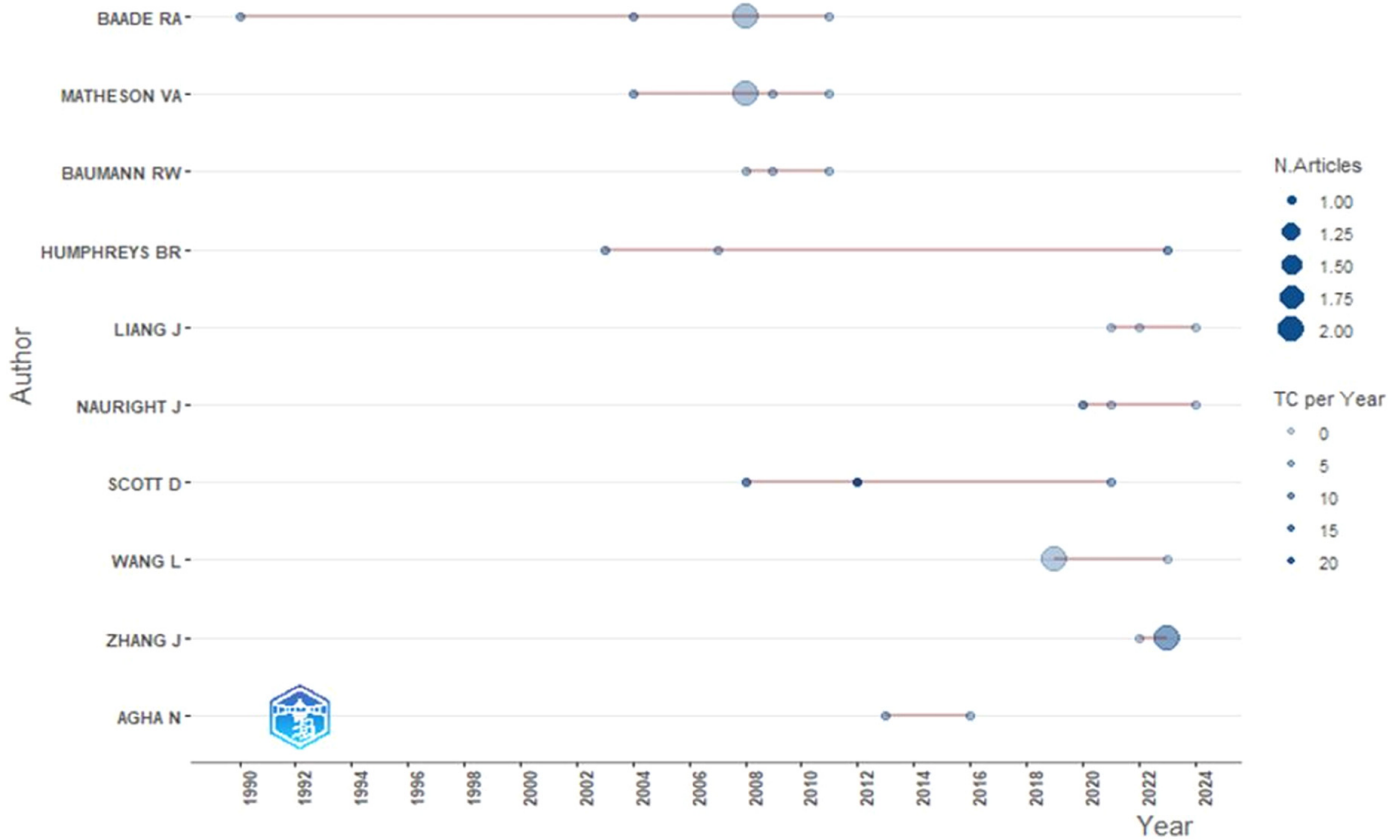
Most influential author's productions over the time 1990–2024.

Figure 4 below shows the productivity of these most influential authors over time. When analyzing the data in the figure, it should be noted that the first studies appeared as early as 1990 and related to Baade (h-index-4, g-index-5), who published articles between 1991 and 2011 and is one of the more productive authors.

Since 2002, there has been an increase in interest in the topic of the impact of sports on the economy. The rise in interest can be attributed to the increased costs of hosting sporting events. As sports events attract resources from public, municipal, and private investors, it was appropriate to assess the effect and return of their use. Studies have been published by Matheson (h-index-4, g-index-5, number of studies—4) between 2004 and 2012 and by Humphreys (h-index-3, g-index-3, number of studies—4) between 2003 and 2023. It should be noted that Baade, Matheson, and Humphreys have the highest number of citations. Agha (h-index-2, g-index-2) started publishing his research from 2012 onwards, later joined by Zhang (h-index-2, g-index-2). The analysis results suggest a strong positive correlation between the number of citations and author productivity.

**Figure 5:**
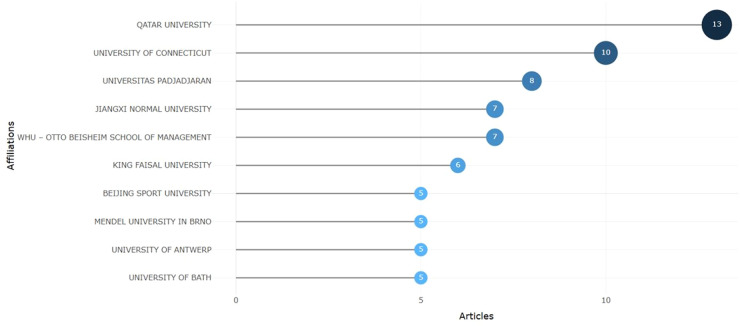
Most relevant affiliations, using R-studio.

Analyzing the affiliation of researchers to specific institutions, the authors conclude that researchers from universities, colleges, and business schools from a vast geography focus on the impact of sports on the economy. Qatar University is the leader with thirteen publications, followed by the University of Connecticut with ten publications and Universitas Padjadjaran with eight publications (see Figure 5).

Qatar University is a public research university located on the northern outskirts of Doha, Qatar. It is the only public university in the country. Qatar University follows the University of Connecticut (USA) with ten publications. The University of Connecticut (UConn) is a public research university. Universitas Padjadjaran is next, with eight publications. Padjadjaran University is a public university located in Sumedang Regency and Bandung, the provincial capital of West Java, Indonesia. In the top ten is Jiangxi Normal University, situated in Nanchang, the capital city of Jiangxi Province, China, with seven publications. It would be helpful to mention here one more university from China, Beijing Sports University, with five publications. Thus, there are two universities from China in the top ten. The seven publications are by WHU (Otto Beisheim School of Management) from Germany and King Faisal University in Hofuf, Saudi Arabia. Spanish universities—University of Granada, University of Zaragoza, University of Cantabria—published a total of 15 articles on the impact of sports on the economy. Five publications are by Mendel University in Brno from the Czech Republic, the University of Antwerp, the University of Bologna from Italy, the University of Oradea from Romania, and the University of Waterloo from Canada.

### 4.3 Sources related metrics

**Figure 6:**
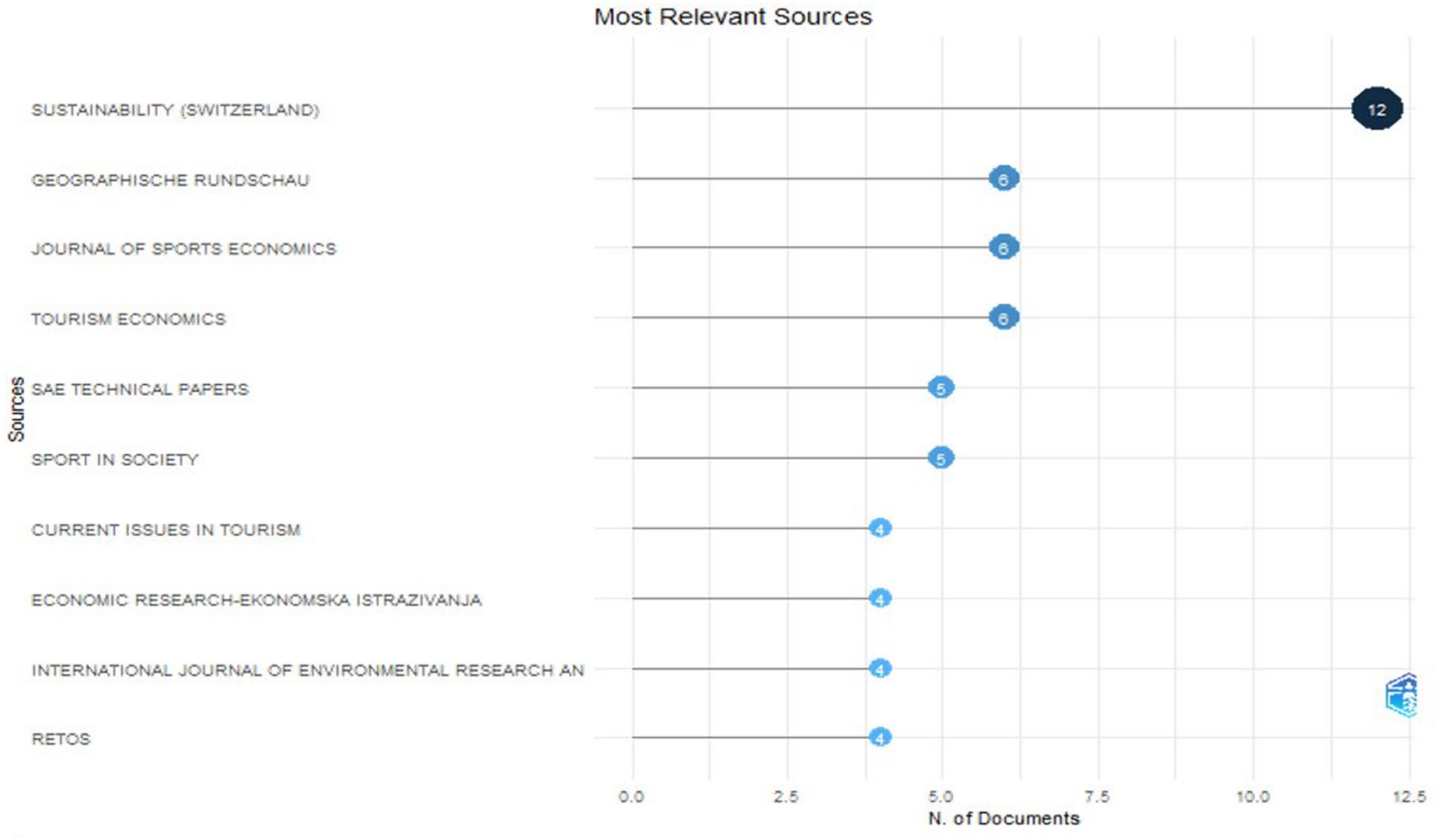
Most relevant sources using R-studio.

The articles analyzed were published in various scientific journals. Using the R-studio software, the authors have selected and analyzed the scientific journals that have published the most significant number of publications on the problem under study (see Figure 6).

The Resurchify (38) website (https://www.resurchify.com) contains the criteria for evaluating scientific journals:

✓ Impact score—indicates the number of times documents published in a journal within the past two years have been cited in the current year;
✓ The h-index shows the productivity or impact measure of the journal;
✓ SCImago Journal Rank (SJR) is a metric that ranks journals based on their average prestige per article.

Table 3 below summarizes the results of the source analysis.

**Table 3:** Most relevant sources (data for 2023).

Name	Impact score	h-index	SJR	Overall ranking	JCR	Number of publications
Sustainability (Switzerland)	3,95	169	0,672	7,863	Q2	12
Geographische rendschau	0,02	13	0,138	23,826	Q1	6
Journal of Sport Economics	2,2	56	0,777	6,505	Q1	6
Tourism economics	5,9	73	1,257	3,056	Q1	6
SAE Technical Papers	0,55	122	0,207	19,579	–	6
Sport in Society	1,86	50	0,534	10,142	Q1	5
Current Issues in Tourism	10,23	108	1,916	1,451	Q1	4
Economic Research-Ekonomska Istraivanja	6,41	53	0,83	5,884	Q2	4
International Journal of Environmental Research and Public Health	3,43	198	0,808	6,138	Q2	4

Created by https://www.resurchify.com.

The table shows that the studies have been published in high-ranking scientific journals with high citation and impact rates.

## 5 Science mapping

Science mapping examines the relationships between research constituents (39). It includes citation, co-citation, and co-authorship analysis.

**Figure 7:**
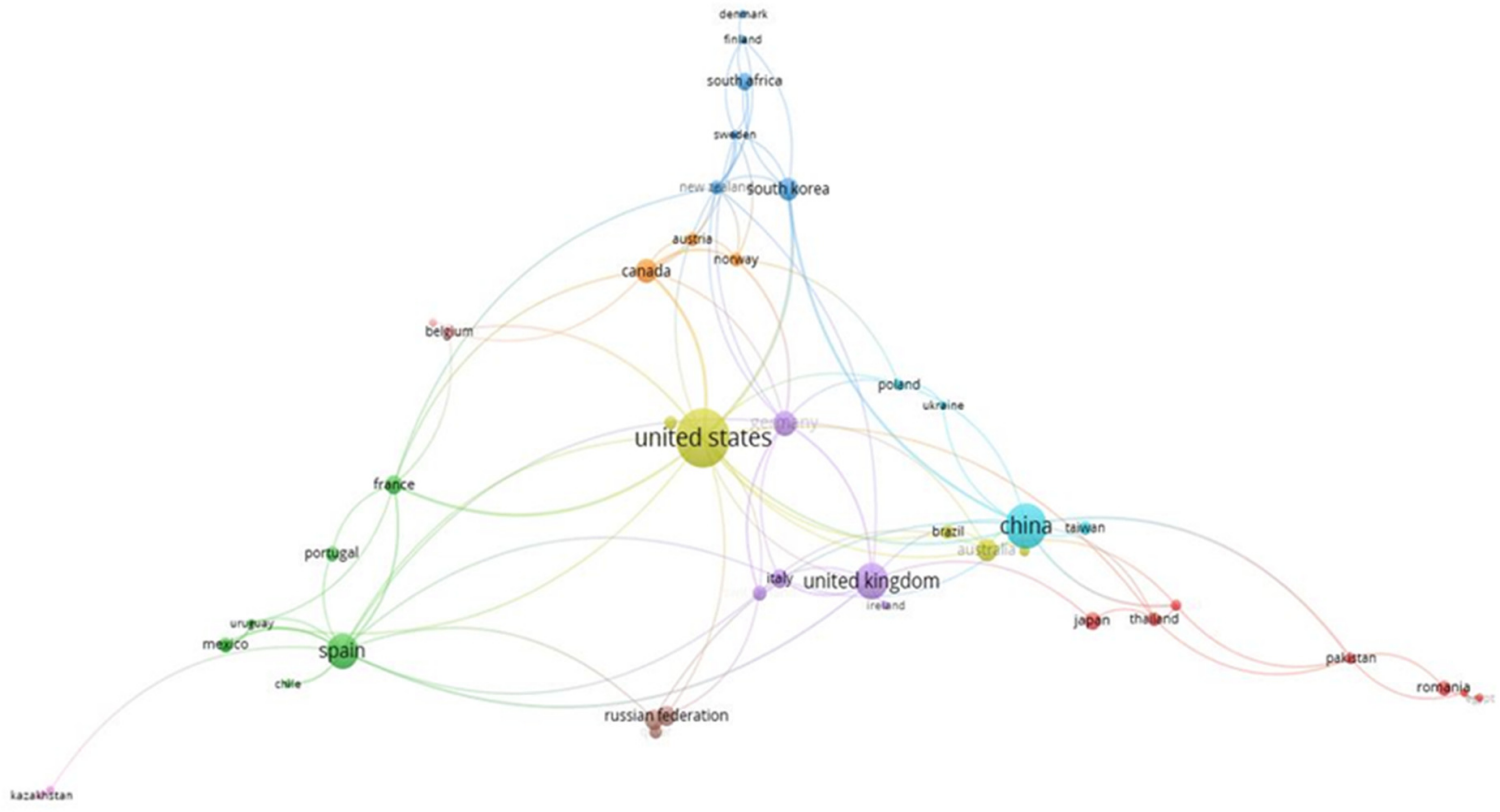
Co-authorship links by country using VOSViewer.

Using VOSViewer, the authors visualized the co-authors’ collaborative links by country (see Figure 7).

The figure shows that US researchers collaborate more actively with researchers from other countries. US researchers have established collaborative links with researchers in virtually all countries in the field of the economic impact of sport. UK, China, and Spain researchers also collaborate with foreign co-authors.

Citation analysis is an essential technique for science mapping. Citation is “one of the most important uses of scientific information in documented scientific communication” (40). Citation analysis allows us to identify relationships between publications, determine the structure of fields of knowledge, and even predict their development. New scientific trends usually emerge within existing ones, so their emergence is reflected in the bibliographic apparatus of publications. Citation analysis allows the identification of established but not yet formally identified strands and possibly new problems, some of which will subsequently develop into independent scientific fields.

**Figure 8:**
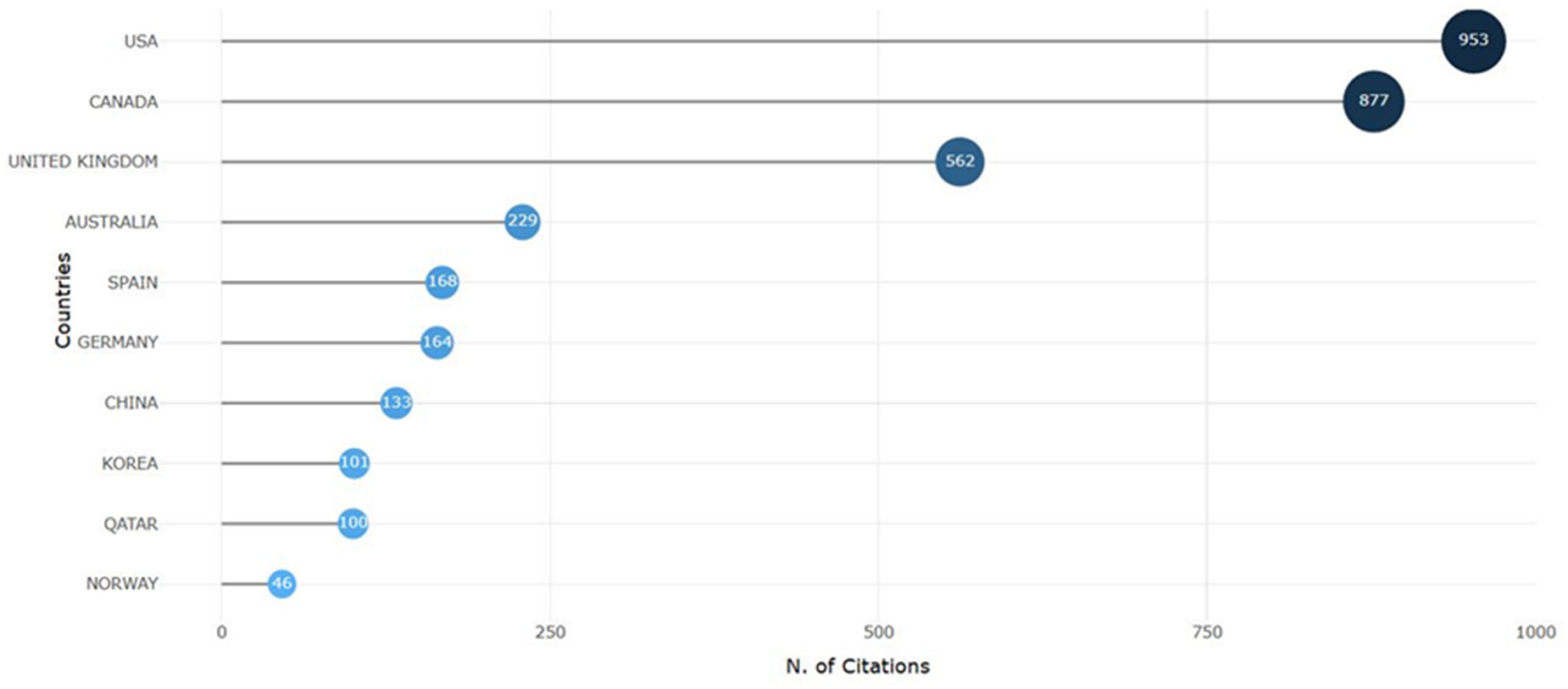
Citation of authors according to R studio.

When assessing citation rates by country (see Figure 8), it should be noted how researchers from the USA dominate, with 952 citations to their research. Canada follows the US with 877 citations and the UK with 577 citations.

Today, international collaboration has become one of the most significant indicators of scientific quality, enhancing both research productivity and impact. Bibliometric analysis indexes allow for the assessment of the level of collaboration. For example, the SCP (Single Country Publications) indicator includes publications authored by researchers from a single country, thus reflecting the internal research capacity of that country. Such publications highlight a nation's ability to conduct independent research and generate original knowledge. The analysis of SCP patterns is particularly important for policymakers and funding agencies seeking to support and strengthen national research initiatives. On the other hand, the MCP (Multiple Country Publications) indicator refers to publications involving authors from multiple countries, indicating active international collaboration and knowledge exchange. This type of cooperation is often associated with higher citation rates and broader dissemination of research findings.

Table 4 exhibits the list of corresponding authors’ countries with their number of publications and an analysis of the number of single-country publications (SCP), multiple-country publications (MCP), and multiple-country publication Ratios.

**Table 4:** Top 10 corresponding authors’ country and publications.

Country	Arcticles	Arcticles %	SCP	MCP	MCP%
USA	46	15,4	36	10	21,7
China	36	12	31	5	13,9
UK	21	7	20	1	4,8
Spain	15	5	11	4	26,7
Germany	9	3	8	1	11,1
Korea	8	2,7	4	4	50
Australia	5	1,7	5	0	0
Canada	5	1,7	4	1	20
Italy	5	1,7	5	0	0
Turkey	5	1,7	5	0	0

**Figure 9:**
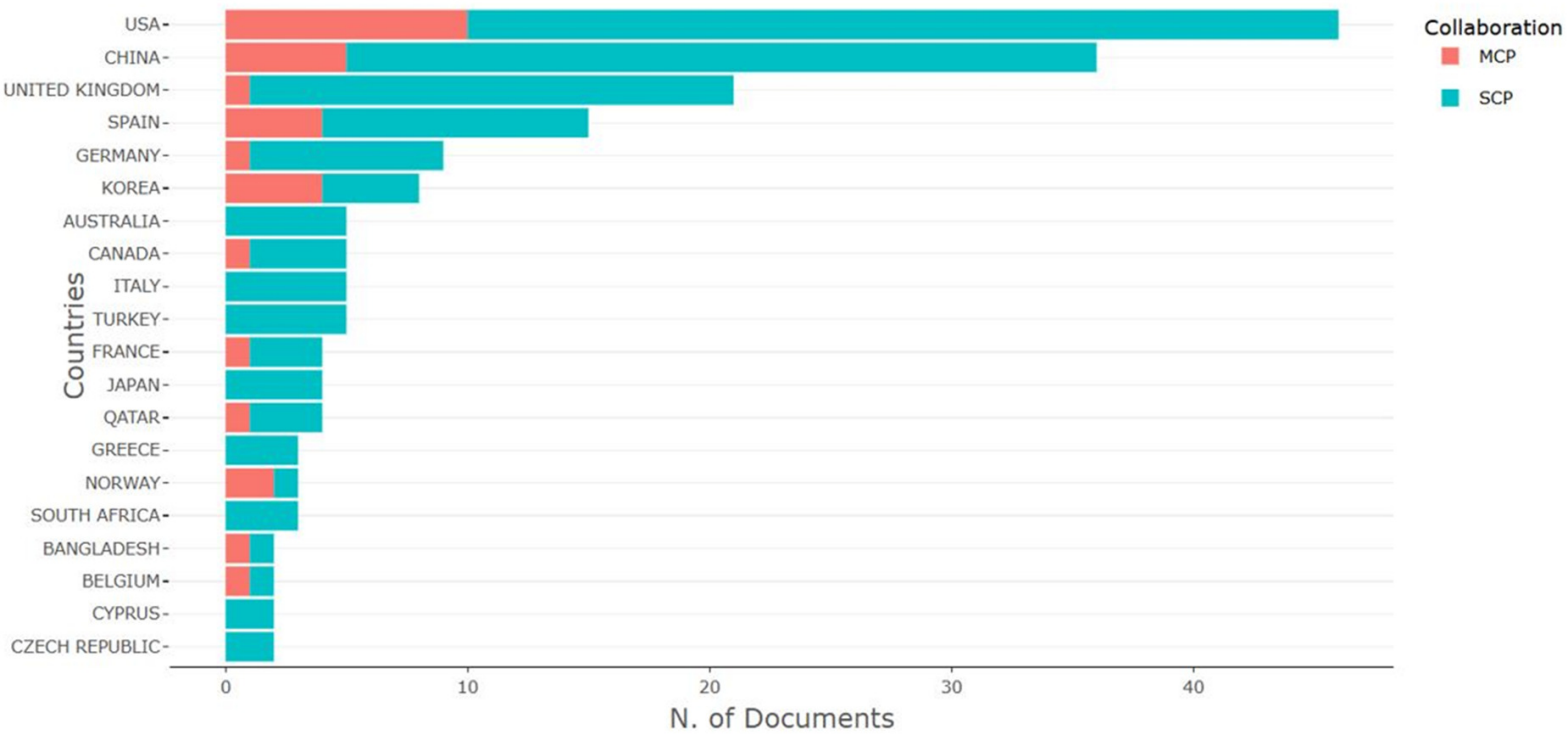
Corresponding author's countries according to the R-programme.

The USA is the leading country with 46 publications, 36 single-country publications, and 10 multi-country publications with an MCP of 0.21. China is in second place with 36 publications; a higher proportion of publications are single-country publications, with an MCP of 0.139. The UK is third with 21 publications, and almost all publications (20) are single-country publications. The high MCP ratio shows the greater collaboration of a country with other countries. Korea has the highest MCP ratio of 0.5. The number of published studies is not high (8 publications), but 4 are single-country publications, and four are multiple-country publications. Figure 9 is the graphical representation of the corresponding authors’ countries and publications.

The figure's analysis shows that SCP publications dominated during the period under study, which means that researchers from the same country collaborated with each other. The exceptions are US and Chinese researchers who involved foreign colleagues in their research. This is a huge problem that needs to be addressed, as cooperation between researchers from different countries will only have a positive impact on the quality of research.

**Figure 10:**
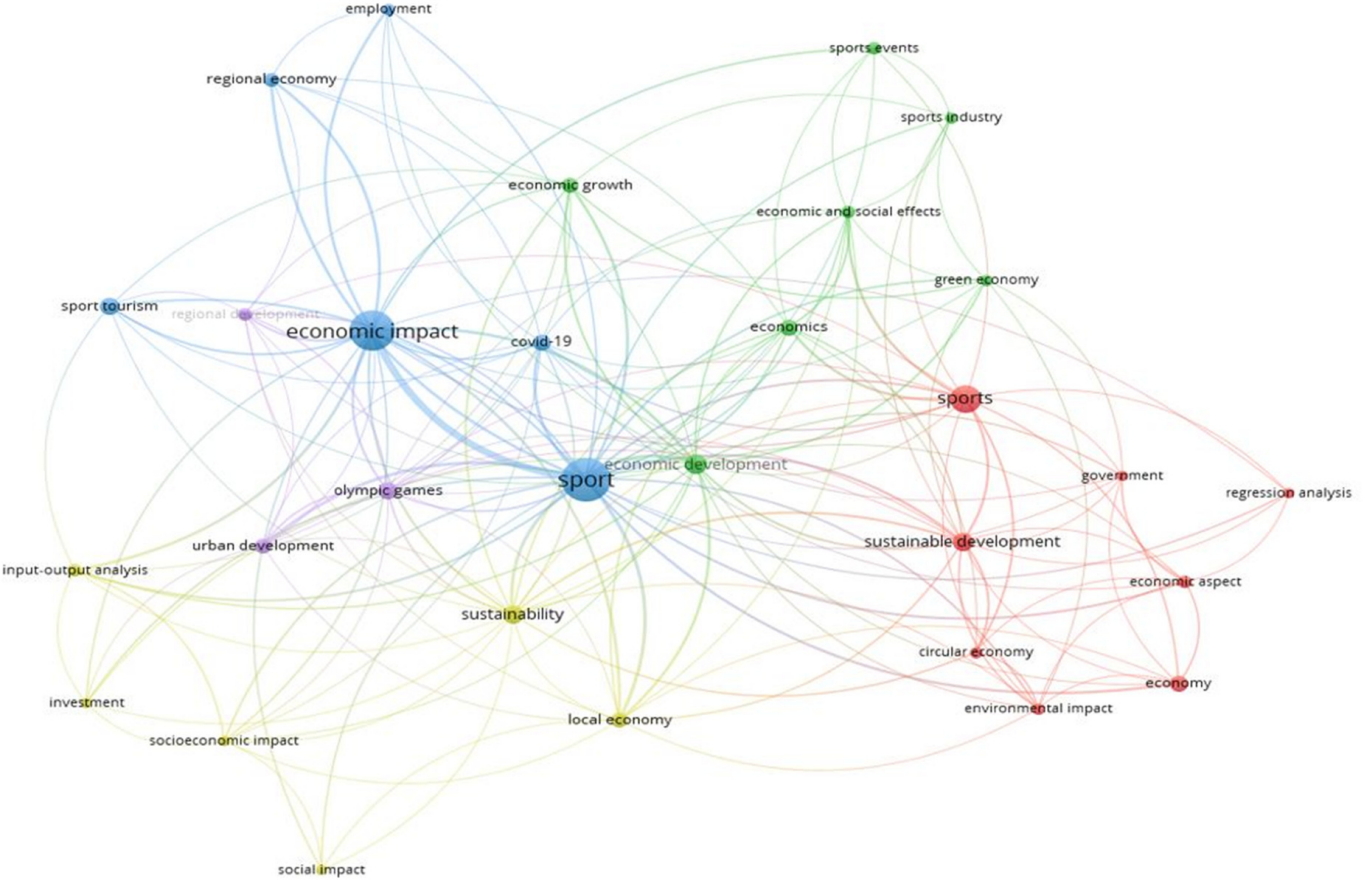
Keywords co-occurence visualization using VOSViewer.

Keywords co-occurrence was visualized using VOSViewer (see Figure 10). Each cluster is represented in a different color; the lines indicate the strength of the relationship between the clusters.

In the figure below, you can see that the most frequent keywords used by authors in studies related to the economic impact of sports are “sport”, “economic impact”, “sustainable development”, “sports tourism”, and “economic aspect”. The figure below shows five clusters. For example, the blue cluster indicates strong links between the keywords “economic impact” and “sport”, “regional economy”, “ employment”, and “sports tourism”, meaning that the authors often use these keywords together, as the economic impact of sports is often seen through the prism of employment and tourism in a regional aspect. The yellow cluster indicates that the socio-economic impact of sports is often assessed through input-output analysis and that sports development is often linked to the sustainable development of the local economy. The pink cluster indicates that researchers are exploring the impact of the Olympic Games on urban development and regional economies. The green cluster indicates that authors often study the impact of the sports sector and “sports events” on national “economic growth” and economic development. The red cluster points out that the keywords “sport” and “sustainable development” are often used in the same study and that when studying the impact of sports on the economy, the authors examine government policies in the field of sport.

## 6 Discussion

Much of the research focuses on the impact of mega sporting events such as the Olympics or World Cups on the economies of the host countries and cities. There is much debate about whether such events deliver sustainable economic growth. Some researchers believe that major sporting events are a golden opportunity for a country to showcase itself to the world while also generating a substantial economic return (41–43), but increasingly, authors of publications are skeptical about the economic impact of such events, concluding that it is too expensive to host mega sporting events in their country, while also citing the disruption to non-participating firms during sporting events as a significant negative factor (20, 25, 44, 45). For example, Baker and co-authors (2020) consider that it may be worthwhile for a country to host the World Cup, but hosting either Olympic Games would likely be costly (39). Our analysis of publication trends confirms that major sporting events remain a pivotal yet contentious theme. There is also scholarly disagreement on the usefulness of new infrastructure, such as whether building new stadiums and sports arenas stimulates local economic growth or becomes a burden on taxpayers. While the organization of major sporting events and the efficiency of infrastructure are debatable issues, scholars are divided on the need to invest in sports and the positive effects of these investments on national and regional economies. Mitchel (48) concludes that sports significantly impact local economies. “Sports helps to create jobs, attract tourists, and boost employee well-being and productivity” (48). The role of sports is irreplaceable within the spectre of national economic growth and regional development (46). Luo proved that the aggregation of the sports industry can increase the level of high-quality development of green energy (47). The positive impact of sports on employment growth, tourism flows, and the efficiency of public funding has been demonstrated (21, 28).

According to the authors’ research, there is a wide range of opinions regarding the economic impact of mega sporting events. A jointly developed universal model (methodology) could facilitate the decision-making process for policymakers, sports institution leaders, and local authorities. This model would enable the calculation and forecasting of both qualitative and quantitative impacts, as well as direct and indirect effects on the overall or regional economy. Such a model would help determine whether a specific sporting event should be implemented.

## 7 Conclusion

Finally, our research findings on the impact of sport on the economy can be taken as an essential indicator for the field's place in scientific literature. However, the number of studies in this scientific field remains limited, indicating a lack of attention by researchers to the importance of the sports sector.

We conclude that there is a trend towards the development of a universal model that would allow not only qualitative but also quantitative impacts on countries, host regions, and individual economic sectors to be measured. This will facilitate the decision-making process for hosting sports events and the costing process, and allow for determining the effect or impact on the economy. In this context, it would be necessary to strengthen cooperation and collaboration between researchers, universities in different countries, sports federations, and responsible public authorities. Sports administrators and researchers need to report to the public on sports’ impact on the economy, publishing the results of both large and small sporting events and risks, thus creating a competitive environment.
